# On Modeling Ensemble Transport of Metal Reducing Motile Bacteria

**DOI:** 10.1038/s41598-019-51271-0

**Published:** 2019-10-10

**Authors:** Xueke Yang, Rishi Parashar, Nicole L. Sund, Andrew E. Plymale, Timothy D. Scheibe, Dehong Hu, Ryan T. Kelly

**Affiliations:** 1 Division of Hydrologic Sciences, Desert Research Institute, Reno, NV 89512 USA; 2 Energy and Environment Directorate, Pacific Northwest National Laboratory, Richland, WA 99354 USA; 3 Environmental Molecular Sciences Laboratory, Pacific Northwest National Laboratory, Richland, WA 99354 USA; 4 Department of Chemistry and Biochemistry, Brigham Young University, Provo, UT 84602 USA

**Keywords:** Motility, Hydrology

## Abstract

Many metal reducing bacteria are motile with their run-and-tumble behavior exhibiting series of flights and waiting-time spanning multiple orders of magnitude. While several models of bacterial processes do not consider their ensemble motion, some models treat motility using an advection diffusion equation (ADE). In this study, *Geobacter* and *Pelosinus*, two metal reducing species, are used in micromodel experiments for study of their motility characteristics. Trajectories of individual cells on the order of several seconds to few minutes in duration are analyzed to provide information on (1) the length of runs, and (2) time needed to complete a run (waiting or residence time). A Continuous Time Random Walk (CTRW) model to predict ensemble breakthrough plots is developed based on the motility statistics. The results of the CTRW model and an ADE model are compared with the real breakthrough plots obtained directly from the trajectories. The ADE model is shown to be insufficient, whereas a coupled CTRW model is found to be good at predicting breakthroughs at short distances and at early times, but not at late time and long distances. The inadequacies of the simple CTRW model can possibly be improved by accounting for correlation in run length and waiting time.

## Introduction

The complex and interacting processes of bacterial transport impart a self-propelling character to many species^1^. The motility pattern is often seen to exhibit an enhanced diffusion^2–8^ with mean-square displacement growing faster than linear in time. In systems with background flow, while immotile cells follow Gaussian-like distributions for velocity and orientations, motile cells have been observed to follow anomalous non-Gaussian deviations^9^. Nevertheless, many models used to study the ensemble behavior of bacterial transport are based on the use of advection-diffusion equation (ADE)^10–15^. The ADE is a manifestation of Fick’s law, valid for uncorrelated velocity fields and characterized by plumes that spread in proportion to *t*^1/2^. Gaussian-like distributions of motion increments is a key assumption of ADE models and hence their use in modeling movement of motile cells may not fully capture the features and trends of bacterial transport. In some cases, use of an ADE-based model to fit the experimental observations of bacterial motion has resulted in inexplicable values of fitting parameters, such as values of retardation coefficient of less than 1^11,12^. This study aims to: (a) help gain more insight into applicability and potential inadequacies of ADE-based models to study bacterial transport, (b) demonstrate construction of simple models for study of ensemble transport by honoring the bacterial species specific motility dynamics, and (c) provide guidance for making further improvement in these modeling techniques.

One of the application areas requiring accurate modeling of motility dynamics is the microbially-mediated reduction of metals and radionuclides. Oxidized forms of many of these contaminants are highly soluble, but form precipitates with lower solubility upon reduction, leading to minimal mobility in the environment^16^. Most numerical models of metal bioremediation treat bacterial biomass as an immobile constituent^17,18^. Field experiments have however observed a strong correlation between the rate of metal reduction and the abundance of planktonic (free-swimming) cells^19,20^, suggesting that microbial transport could impact spatial patterns of metal reduction. As a first step in an effort to develop improved simulators for metal bioremediation, we studied motility characteristics and construct simple micro-scale transport models for two microorganisms: the model metal-reducing bacterium *Geobacter sulfurreducens* (strain PCA, the type strain of the species) and *Pelosinus* strain JHL-11, an organism isolated from the uranium- and nitrate-contaminated groundwater of the 300 Area, and chromium contaminated groundwater of the 100 Area, of the U.S. Department of Energy’s (DOE’s) Hanford Site, in southeastern Washington State^21^. *Geobacter* species have been extensively studied and characterized in relation to uranium bioremediation, in particular at the DOE’s Rifle, CO, field research site^22,23^. Metal and radionuclide reduction by *Geobacter* is usually associated with reduction of natural iron oxide minerals, which being much more abundant than the contaminants serve as the primary electron acceptor for *Geobacter* metabolism^24,25^. *Zhao et al*.^26^ developed a numerical model of uranium bioremediation that includes terms describing the bulk movement (passive advection) of planktonic bacteria as well as their attachment to solid surfaces (transition from planktonic to attached phase), and demonstrated through sensitivity analyses that these processes play a significant role in determining the overall rate of contaminant reduction. *Pelosinus* species have been seen to have uranium and chromium reducing capabilities^21,27,28^ and their strains isolated from chlorinated solvent-contaminated groundwater have been observed to express flagellar motility^29^.

Though many metal reducing bacteria are motile with their run-lengths (jump length) and waiting-time spanning a wide range of values, existing models of contaminant bioreduction do not account for the movement of microorganisms, either passive movement with flowing groundwater or active movement by motile (and sometimes chemotactic) bacteria. Accurate numerical models of bioremediation are needed to support design and evaluation of field implementations. We present here experiments and models using motile strains of *Geobacter* and *Pelosinus* to quantify their motion properties and ensemble transport in unobstructed medium, as a prelude to development and testing of new models of bacterial transport during bioremediation.

## Methods

A sequence of experiments was conducted to quantify the two-dimensional movement patterns of individual cells in micro-models in the absence of flow. *Geobacter sulfurreducens* strain PCA^30^ was cultured anaerobically in *Geobacter* Medium (ATCC medium 1957), with sodium acetate at 10 mM and sodium fumarate at 50 mM. Cells were used either without dilution or after diluting in anoxic phosphate-buffered saline (PBS) containing 10 mM sodium fumarate. Although expressing flagellar motility when originally isolated, including chemotaxis proteins^31^, the *Geobacter sulfurreducens* type strain used here (PCA) has lost flagellar motility over time in laboratory incubation. However, the strain retains type IV pili^32^, which can give rise to twitching or gliding motility^33^. Although pili-enabled twitching motility in this strain has not been documented or quantified, to our knowledge, the results of Spears *et al*.^32^ suggest that the pili of *Geobacter sulfurreducens* (strain PCA) may be involved in twitching motility in addition to electron transfer and biofilm formation. *Pelosinus* strain JHL-11, isolated under nitrate-reducing conditions from sands incubated from Hanford Site 300 Area groundwater was cultured anaerobically in tryptic soy broth (TSB), without dextrose, with 5 mM potassium nitrate added as electron acceptor, at either 30 °C or room temperature. Transmission electron microscopy imaging (see Supplementary Fig. 1) suggests that strain JHL-11 contains peritrichous flagella consistent with observations of its close relatives^29^. Such extracellular structures should give rise to swimming motility^34^.

### Experimental setup

**Figure 1:**
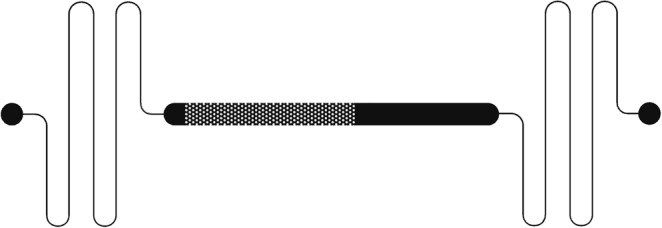
Sketch of the micro-models. The black dots at left-end and right-end are the inlet and outlet which were used for solution injection but were sealed afterwards to ensure no flow. The main body of chamber was divided in an open unobstructed area (black zone in the diagram) and an area with staggered cylinders.

Tracks of cells on the order of several seconds to a few minutes in duration were recorded to provide information on motility in two dimensions. The approach is similar to some other studies where the relatively small value of vertical dimension compared to the extent of horizontal plane allows for projecting the bacterial motion onto two-dimensional planes^13,35–37^. Micro-model chambers for easy injection and viewing of the cells were constructed as shown in Fig. 1.

The non-flowing chambers, made out of polydimethylsiloxane (PDMS) for easy design adaptation, were 20 µm deep in the vertical direction and about 2 mm in the transverse direction. The chamber was divided into an open unobstructed area, and an area designed with staggered cylinders to model a simple pore network. This paper only presents results obtained from the open zone. The chambers were viewed by microscopes in vertically downward direction, limiting the recorded trajectory information to a 2D plane. To limit oxygen exposure to the bacteria, the micro-models were stored in an anaerobic chamber and then kept in anaerobic jars until immediately before use, at which point the anaerobic bacterial suspensions were injected into the micro-model via N_2_-sparged needle and syringe. After injection of the bacterial solution, the inlet and outlet were sealed to eliminate the possibility of any flow affecting active bacterial swimming. The circuitous path leading from the inlet/outlet point to the micro-model chamber was designed to minimize the chances of any small perturbation in pressure gradient to cause flow in the chamber. Bacterial motion was viewed under various magnifications, and videos were recorded using a camera with a sensor pixel size of 13 µm × 13 µm. Each recorded frame was of the size of 1024-pixels × 1024-pixels. The physical size of each pixel on the video file is easily determined by dividing the sensor size by the magnification. For example, with a microscope magnification of 20X, each pixel on the video file has a size of 13/20 = 0.65 µm. The total size of the viewing window then becomes 1024 × 0.65 = 665 µm.

In contrast to study of motility near surfaces which allows for careful examination of single-cell motility mechanisms^38^, viewing and recording bacterial motion in open medium are mainly suited for collecting single-cell trajectory data and also involves more careful adjustments of microscope magnifications. Selection of the microscope magnification was mainly guided by the average body length of the cells being studied. Each individual cell should ideally occupy at least 2–3 pixels on the video frames in order to minimize the numerical issues associated with tracking. For example, a good choice of magnification to observe bacteria with an average body length of 0.5 µm is at least 50X (each pixel on the video file is then 13/50 = 0.26 µm). However, increasing the magnification also results in reduction of the size of viewing window as well as reduction in depth of field, causing the cells to frequently go in and out of the focal plane. Microscope magnification of 20X, 32X, 40X and 64X were used in this study for the two species of bacteria. The small body size of *Geobacter* warrants larger magnification of 40X or 64X, whereas videos of *Pelosinus* were recorded under bright light with 32X and 20X magnification factor. The sampling rates, which have been shown to be an important factor in determination of statistical properties of motility^39^, were varied only within a small range. Videos of the two species were recorded at frequencies (frame capturing speed) ranging from about 4 Hz to 8 Hz to produce files which were sufficiently long and at the same time avoided dramatic changes in location of individual cells from one frame to the next. A large number of video files were collected for both species by repeating the experiments under identical conditions.

### Video processing

A series of video file extraction and processing codes were written in MATLAB to analyze the recorded files created by the ImageJ processing program. Each video file consisted of 1000 frames with every frame being 1024-pixel × 1024-pixel in size. A frame capturing frequency of 8 Hz for example generates a total video duration of 1000/8 = 125 seconds. Pixels appear as a shade of black, white, or gray and are associated with an intensity value. The data of pixel intensity were recorded and read in MATLAB and treated as a 1024 × 1024 × 1000 matrix. Generally, the bacteria are darker than their surroundings under bright light, thus giving the cells a pixel intensity value distinct from their surroundings. The background pixels tend to have similar intensity values in time while the pixels occupied by bacteria change their intensity over time. The method used here is similar to the recently applied image analysis techniques by *Liang et al*.^37^ where individual cell locations were determined by finding the maxima of local intensity in an analyzed image.

Processing of video files to determine trajectories of individual bacteria presents several challenges because of file processing complexities related to shifts in pixel intensity, changes in bacterial swimming speed, and possible intersection of two trajectories. A “moving bacterium” is recognized as an assembly of points, where (1) the intensity of these points is significantly different (darker or lighter) from surroundings in a frame, and (2) the center of these points are changing through time. The x- and y-coordinates were found by locating the centroid of all occupied pixels by a cell. To accurately determine the positions of bacteria and create trajectories, spatial and temporal filters were introduced to eliminate the noise of background. A radius around each path end was defined, outside of which a detected bacterium was considered to be ‘new’. In addition, an appropriate searching radius was created and applied for finding the new location of bacteria after a run (also called jump). Alternative methods for extracting trajectories have been reported in recent literature by finding an optimal association between points in subsequent frames based on maximization of total likelihood of all trajectories^37^. Though comparing the results from different trajectory generation algorithms is a useful exercise, we focus here on statistical analysis of trajectory data and using it to construct ensemble transport models. A movie showing identification and tracking of *Pelosinus* cells in shown in the Supplementary Materials.

### Statistical analysis

After path lines for each bacterium were created, data were further filtered to only include those paths that have meaningful duration (10 frames or more) and at least one jump during the duration of its total recorded time (i.e., bacteria that were completely idle were excluded from further analysis). A cell was assumed to be in “waiting state” if it moved less than the average body length for its species. Continuous motion of a cell in a certain direction was treated as “long” jump unless the direction of motion at some instance changed by more than 5°. A new jump is registered when at least one of two conditions is met: (1) the bacterium moves from a waiting state; or (2) the change in turn angle in the direction of motion is larger than ±5° (about 1 pixel change in perpendicular direction over 10 pixels of displacement in the direction of travel).

As individual trajectories were assumed to be independent of each other, the initial location (i.e., start point of a trajectory) can be arbitrarily shifted in post-processing to make all trajectories start from a common point (for example, the origin of the coordinate system). This is to say that the exact start point and end point of a trajectory within the viewing window doesn’t matter; what matters is the change in pixel location and the time elapsed for those changes to happen. After transferring the time and distance from units of frame and pixels to units of seconds and µm by equations:

$$\begin{array}{rcl}{\rm{Time}}\,(\sec ) & = & {\rm{Number}}\,{\rm{of}}\,{\rm{Frames}}\times (1/{\rm{Frequency}})\\ {\rm{Distance}}\,(\mu m) & = & {\rm{Number}}\,{\rm{of}}\,{\rm{Pixels}}\times (13\,\mu m/\mathrm{Magnification}\,{\rm{Factor}})\end{array}$$

Each individual trajectory of a species was merged into one master file which contains information for all jumps including x-increment, y-increment, and amount of waiting time. This master file was then used to analyze distributional properties of waiting time and jump length for each species.

In addition to recording the statistics of jump length and waiting time, the number of cells in the system, the mean travel distance, and the mean-centered standard deviation of cell locations as a function of time were also continuously monitored. The master file containing information of all trajectories (longer than or equal to 10 frames) progressively becomes sparse as only a few trajectories were continuously recorded for a long duration (e.g., more than 100 frames). This is because a majority of the trajectories go in and out of the viewing window, turning them into discontinuous pieces of data. Mean and variance were computed only up to the time where the master file contained at least 50 unique trajectories.

### Model development

Considering the ‘run-and-tumble’ nature of microbe movement^40^, the Continuous Time Random Walk (CTRW) approach is a promising mathematical framework to model and predict the motion of microbes^41^. The CTRW approach assumes the number of jumps (n) and their magnitude during any given time interval (0, t) to be random. Individual trajectories were considered to comprise single displacements whose length is a random variable and are also separated in time by random waiting periods. A bacterium starting from location **r**_**0**_ jumps to **r**_1,_ and then waits for time τ_1_ before the next jump. Particles are tracked through a series of jumps and waiting periods to express the n + 1 step displacement as:

1 $${{\boldsymbol{r}}}_{{\boldsymbol{n}}+1}={{\boldsymbol{r}}}_{n}+{{\boldsymbol{\varepsilon }}}_{n}$$

where **ε**_n_ is the displacement increment at step n + 1. The n + 1 step time can be expressed as:

2 $${{\rm{t}}}_{{\rm{n}}+1}={{\rm{t}}}_{{\rm{n}}}+{{\rm{\tau }}}_{{\rm{n}}}$$

where τ_n_ is the time increment at step n + 1. In this study, displacement (**r**_i_) and waiting time (τ_i_) are modeled as both coupled and uncoupled, by treating waiting time as dependent variable (i.e., waiting time increment is computed by first conditioning the random walk process on the jump length) or as an independent variable (i.e., waiting time and jump length are treated as unrelated quantities) respectively. The displacement increment **ε**_n_ and the time increment τ_n_ are directly derived from the empirical probability density functions of jump length and waiting time formed by extracting those data from trajectories of individual cells. For the coupled model, specific waiting time values that correspond to known jump lengths are used in the random walk process. For the uncoupled model, all possible values of waiting time are treated as equally likely regardless of the magnitude of jump length. Let P (t; n) be the probability for n jump events in time t. P (**r**, t), the probability of finding the particles at **r** at time t, can be expressed as^42^:

3 $${\rm{P}}({\bf{r}},{\rm{t}})={\sum }_{{\rm{n}}=0}^{\infty }{\rm{P}}({\rm{t}};{\rm{n}}){{\rm{P}}}_{{\rm{n}}}({\bf{r}})$$

where P_n_(**r**) is the probability of finding the particle at **r** after n jumps. The computation of mean travel distance and mean centered variance from recorded trajectory data allows for construction of an advection diffusion equation (ADE) based model, which has been used by several researchers to study bacterial transport^7,11,12,43–45^. The general form of ADE can be expressed as:

4 $$\frac{\partial {\rm{C}}}{\partial {\rm{t}}}=-\,\nabla \cdot ({\bf{v}}{\rm{C}})+\nabla \cdot ({\rm{D}}\nabla {\rm{C}})$$

where C is concentration of particles, **v** is flow velocity, and D is diffusion tensor. At the foundation of the ADE lies the assumption that the variance of migrating particles grows linearly with time, a characteristic of Fickian diffusion. In many circumstances however the dynamics of the migrating particles can either suppress or enhance the rate of diffusion to give rise to sub-Fickian or super-Fickian phenomenon. This can be accounted for in the ADE model by allowing the diffusion coefficient, D, to vary in time and is computed as:

5 $$D=\frac{1}{2}\times \frac{\partial {{\sigma }}^{2}}{\partial t}$$

where σ is standard deviation of distance traveled. With the self-propelling nature of their motion, it is intuitive to expect motile bacteria to exhibit non-Fickian behavior. All bacterial species are however not alike in their motion characteristics and it is possible that the growth of variance with respect to time may span a wide spectrum of behaviors for various species.

**Figure 2:**
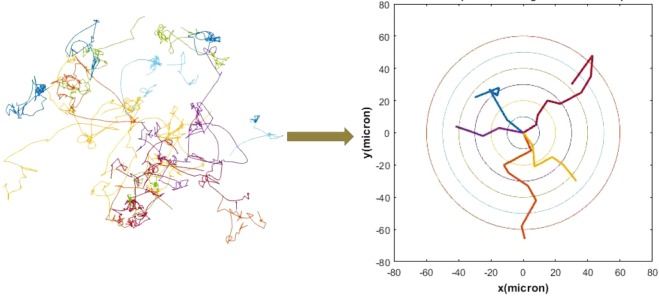
Illustration of computed trajectories (set of 20) and control plane settings. The concentric circles represent control planes located at 10, 20, 30, 40, 50, and 60 µm away from point source (0, 0). The starting points of all trajectories are moved to the origin and their first passage times (breakthrough plots) are recorded for different control planes.

The raw trajectory data can be replicated to reproduce the real observed ensemble transport. This establishes the “true” breakthrough profile that ideally should be reproduced by a robust model. The goal of the model development here is to compare the predicted breakthrough plots obtained from the CTRW (both coupled and uncoupled) and ADE model with that of the observed transport and analyze the successes and shortcomings. All recorded trajectories were translated such that they start from the origin and diffuse radially to concentric rings of control planes located at fixed radial distance (see Fig. 2). The breakthrough plots, which in essence are the first passage time densities, as bacteria can move back and forth multiple times across a control plane, are computed and compared.

The duration of the longest recorded video was about 250 seconds (1000 frames recorded at a low frequency of approximately 4 Hz), hence the breakthroughs are limited in time by that value. The modeled breakthrough plots obtained using CTRW and ADE models can however continue up to any desired value in time. For the CTRW model, random values were generated for each step for magnitude of jump length and waiting time from pre-determined probability density functions. For the coupled model, the jump length and waiting time are dependent. For the uncoupled model, jump length and waiting time are selected independently of each other. Breakthrough plots at L= 10, 20, 30, 40, 50, and 60 µm were obtained by both coupled and uncoupled model for *Pelosinus*, and only at the first four control planes for *Geobacter*. To obtain breakthrough plots using the ADE model, solution to the 1-D ADE equation was considered^46^:

6 $$\frac{{\rm{C}}}{{{\rm{C}}}_{0}}=\frac{1}{2}{\rm{erfc}}(\frac{{\rm{L}}-{\rm{V}}\times {\rm{t}}}{2\times \sqrt{{\rm{D}}\times {\rm{t}}}})\,$$

where C is the number of bacteria reaching distance L in time t, C_0_ is the initial number of bacteria, L is distance to the control plane, V is the average velocity, which is obtained from slope of mean displacement over time, and D is the diffusion coefficient, obtained using Eq. (5). Note that the value of D changes with time as the rate of growth of variance is not uniform. For time periods exceeding the maximum duration of recorded trajectories, the diffusion coefficient is assumed to be a constant (equal to the value of D computed for the longest recorded trajectories).

## Results

### Jump length and waiting time

**Figure 3:**
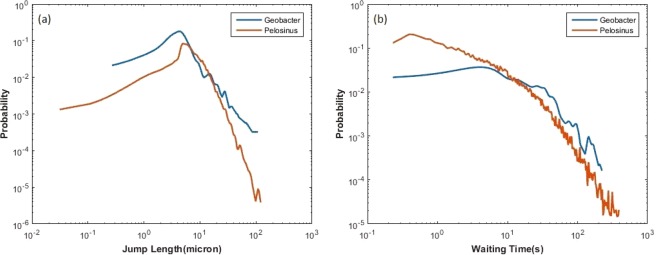
Jump length and waiting time probability densities for (**a**) *Geobacter* and (**b**) *Pelosinus*.

The empirical probability density functions (PDF) of jump length and waiting time for the two species are shown in Fig. 3. The probability densities of the jump length show the same trend for the two species: the probability increases until it reaches the peak (5–6 µm), after which it decreases with a maximum recorded jump value of about 100 µm. The longest jump of 100 µm is about 20 to 50 times of bacteria body length. For *Geobacter*, with pili enabled twitching motility, the PDF of jump lengths spans a shorter range and shows a higher probability associated with longer jumps compared to that of *Pelosinus*. In contrast, for *Pelosinus*, with flagella driven swimming motility (see Supplementary Fig. 1), the PDF of jump lengths spans a wider range and shows a lower probability associated with longer jumps when compared to *Geobacter*. The waiting time PDF for *Geobacter* has a high and nearly-constant probability for low values of waiting time (<10 seconds), after which the probability gradually declines. The waiting time PDF for *Pelosinus* continuously declines and shows a higher probability associated with shorter waiting periods and lower probability associated with longer waiting period compared to *Geobacter*. Looking at Fig. 3, one can say that *Pelosinus* is more likely to make fast jumps and less likely to make slow jumps when compared with *Geobacter*. The longest waiting time can be over 200 seconds for both species.

### Trajectory analysis

**Figure 4:**
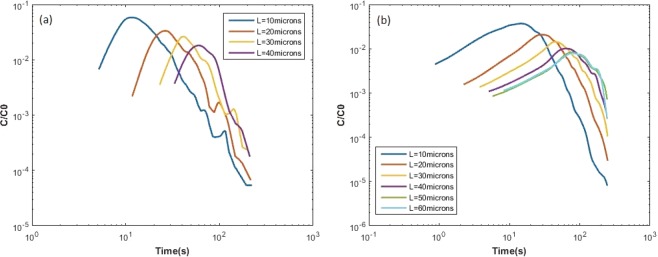
Normalized first-arrival –time curves (breakthrough plots) at various control planes for (**a**) *Geobacter* and (**b**) *Pelosinus*.

The first-arrival-time curves of *Geobacter* and *Pelosinus* are shown in Fig. 4. These were computed by tracking the real trajectories, repositioned to begin at the origin, until the control planes were reached. These are the “true” trajectories that models are expected to match. For *Geobacter*, only about 10% of bacteria reached the first control plane (L = 10 µm) within the available maximum duration of recorded videos. In the case of *Pelosinus*, more than 30% of the bacteria reached the first control plane. The recovery rates of *Pelosinus* reaching every control plane are higher than that number of *Geobacter*, which indicates that *Pelosinus* is generally more “active”. For L = 50 and 60 µm, the recovery of the *Geobacter* was lower than 2% and it does not display a clear profile of breakthrough. For this reason, the breakthrough plots for *Geobacter* were not computed for L = 50 or 60 µm. As control planes become farther, the peak time of curves migrate to higher values and the magnitude of peak concentration decreases. For *Pelosinus*, the curves have a larger spread and a longer rising limb, while *Geobacter* has narrower spread and a steeper rising limb.

**Figure 5:**
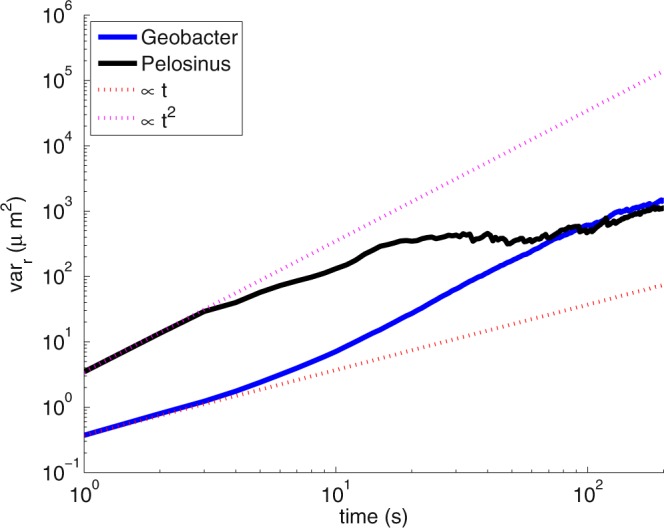
Growth in spreading of location of cells as a function of time.

Figure 5 shows the plots of variance (mean-centered second moment) in location of the two species over time providing valuable insight into the possibility of non-Fickian transport behavior. On the log-log plots of Fig. 5, variance exhibits a linearly increasing relationship with respect to time for *Geobacter*, and for *Pelosinus* the variance increases at a much faster rate (i.e., super-Fickian behavior) at early times. The rate of growth in spreading increases with time for *Geobacter* while it reduces with time for *Pelosinus*. For *Geobacter*, the approximate Fickian behavior of transport, at least at early times, raises the possibility that an ADE based model might perform better in comparison to *Pelosinus*.

Though experiments were performed carefully to prohibit any movement of fluid inside the micro-model chambers (by designing circuitous inlet and outlet paths for added back pressure and by sealing the chamber after injection) our analysis showed that the mean position of the ensemble of particles did move at a very gradual pace (see Supplementary Fig. 2), This could be caused by an improper sealing of the chamber or by a minute tilt or vibration of the experimental apparatus. In our experiments, the mean displacement of *Geobacter* moved at a speed of 0.22 µm/s and that of *Pelosinus* moves at a speed of 0.15 µm/s. These values, though very small, were included in the ADE model computations.

### Model comparison

**Figure 6:**
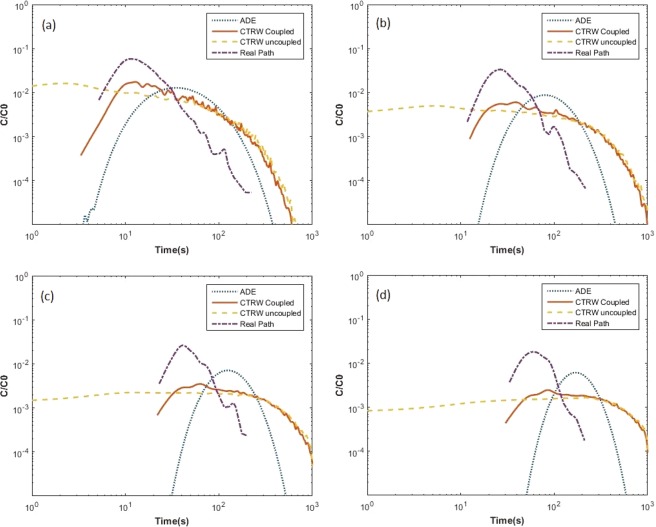
Comparison of real breakthrough plot and modeled breakthroughs for *Geobacter* at (**a**) L = 10, (**b**) L = 20, (**c**) L = 30, and (**d**) L = 40 µm.

Figure 6 shows breakthrough results obtained from real path, ADE model, coupled CTRW model, and uncoupled CTRW model for *Geobacter* at L = 10, 20, 30, and 40 µm. The uncoupled CTRW results do not show clear peaks, have very wide spread, and predict a high concentration for very small values of time. The coupled CTRW model and the ADE model performed relatively better than uncoupled CTRW in approximating the “real breakthrough”. The modeled results generally perform poorly for all control planes in the case of *Geobacter*. The breakthrough plots of real path show a reduced spread and rapid rising and falling limb when compared to the modeled results. Coupled CTRW performs better at predicting early transport and matching the time to peak. However, when it comes to late time, coupled and uncoupled CTRW models yield similar curves, which decline significantly slower than real path breakthrough plots. The ADE results for *Geobacter* in fact show a closer match with the real path breakthrough at late times. The better performance for ADE (at least for late time) is not totally unexpected as the plot of variance with respect to time for *Geobacter* shows a closer trend to a linear relationship (Fig. 5).

**Figure 7:**
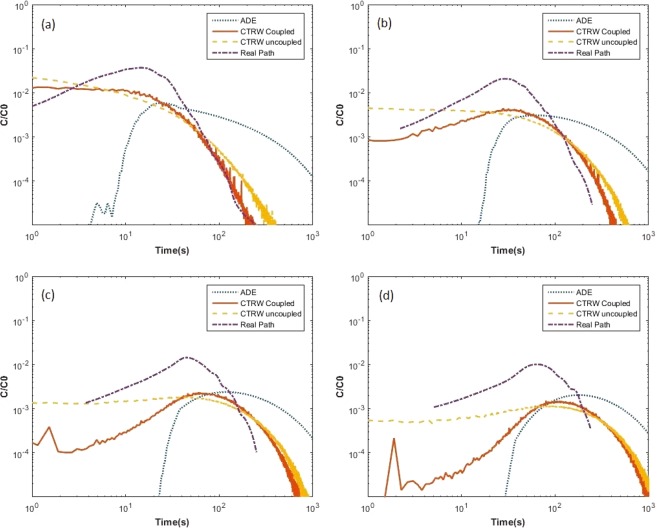
Comparison of real breakthrough plot and modeled breakthroughs for *Pelosinus* at (**a**) L = 10, (**b**) L = 20, (**c**) L = 30, and (**d**) L = 40 µm.

Figure 7 shows comparisons of breakthrough plots obtained from different models and real path for *Pelosinus* at L = 10, 20, 30, and 40 µm. The recovery rates for L = 50 and 60 µm were very small (~3%) and hence those plots are omitted from Fig. 7. The ADE performed very poorly on all aspects of breakthrough attributes (i.e., shape, spread, peak magnitude, or rate of rise and decline). Coupled CTRW model performs well in matching the real path breakthrough plots at shorter distance control plane. The performance of the CTRW models becomes poorer for longer control plane distances. The uncoupled CTRW model agrees with the coupled CTRW fairly well at late times.

Tables 1 and 2 list the mean values and standard deviations of real path breakthroughs and modeled breakthroughs for *Geobacter* and *Pelosinus*, respectively. The mean values of all three models (coupled and uncoupled CTRW, and ADE) for *Geobacter* are 3 to 5 times larger than the mean values of real path. The mean-centered standard deviations of ADE models are significantly lower than CTRW models for *Geobacter*, pointing to relative suitability of an ADE-based model (compared to CTRW models) for studying this species’ ensemble transport behavior. For *Pelosinus*, the ADE-based model performs very poorly. Both the coupled CTRW and uncoupled CTRW models better approximates the real path mean and standard deviation for *Pelosinus* than in the case of *Geobacter*. The performance of CTRW models gets weaker with increasing distance of the control planes. This may imply that successive space and time steps for individual bacterium are not independent from one another. The reduction in performance of CTRW models is more pronounced in predicted values of mean; the predicted values of mean-centered standard deviation in fact improves (i.e., becomes closer to the computed values using real path) with increasing distance of the control planes.

**Table 1:** Mean and Standard Deviations (STD) of each modeled breakthrough plot for *Geobacter*.

	Real Path		ADE		Coupled CTRW		Uncoupled CTRW	
	Mean^a^	STD^b^	Mean	STD	Mean	STD	Mean	STD
L = 10 µm	23.03	21.57	69.67	47.69	84.45	86.36	95.15	100.05
L = 20 µm	41.43	25.68	115.13	58.09	187.80	161.99	199.53	177.21
L = 30 µm	58.59	29.91	160.58	66.90	279.87	209.28	291.75	223.56
L = 40 µm	72.44	29.44	206.04	74.68	353.97	233.85	363.17	247.06

^a^In unit of sec, ^b^In unit of sec.

**Table 2:** Mean and Standard Deviations (STD) of each modeled breakthrough plot for *Pelosinus*.

	Real Path		ADE		Coupled CTRW		Uncoupled CTRW	
	Mean^a^	STD^b^	Mean	STD	Mean	STD	Mean	STD
L = 10 µm	22.33	17.81	286.18	318.03	24.95	26.17	30.10	38.47
L = 20 µm	45.90	31.15	350.90	336.62	69.67	51.21	69.80	59.30
L = 30 µm	68.17	41.57	412.66	354.01	109.37	58.87	100.94	66.10
L = 40 µm	88.32	47.72	476.74	370.32	137.34	57.37	122.44	66.73
L = 50 µm	105.48	54.57	541.68	385.20	157.41	54.24	137.47	65.45
L = 60 µm	104.85	51.76	606.50	398.72	173.20	49.00	148.60	63.64

^a^In unit of sec, ^b^In unit of sec.

## Discussion

*Geobacter* and *Pelosinus* are commonly known metal reducing microorganisms with pili enabled and flagella driven swimming motility respectively. It has been reported that pili are important for both motility and metal reduction capacity of *Geobacter*^47^ and the motility has been observed to be an important factor for *Geobacter* in bioremediation^48^. Similarly, *Pelosinus* isolated from heavy metal contaminated areas of the Hanford Site^21^, has been reported to prefer a planktonic state rather than sediment-attached state in studies of reductive chromium immobilization in flow-through column experiments^49^.

The jump length and waiting time probability density functions of *Geobacter* and *Pelosinus* reveal the nature of transport of these two species. Both the jump length and waiting time probability densities have heavy tails, which suggests that these two species of bacteria depart significantly from the Brownian motion type random walk processes. The jump length was found to exceed 100 µm, which is 20–50 times of the body length of these two species of bacteria^30,50^. The zeroth moment (recovery rates) of breakthrough plots suggests that *Pelosinus* is more active than *Geobacter*.

Data were collected in the form of video files, with each file consisting of information on multiple independent trajectories. MATLAB analysis of the video files resulted in 6827 independent *Geobacter* trajectories and 20226 independent *Pelosinus* trajectories. For *Geobacter*, the real path data consisted of trajectory runs ranging from 0.4 s to 4.8 s, and for *Pelosinus*, the real path data consisted of trajectory runs ranging from 0.4 s to 37.6 s. *Geobacter* exhibited a linear relationship between variance and time in the initial phase (t < 10 sec) whereas *Pelosinus* showed a strong super-Fickian behavior at early times.

First-arrival time plots (breakthrough plots) of real path have relatively sharp peaks and narrower spread than ADE and CTRW modeled results. The ADE results show that the model may come close to approximating *Geobacter* ensemble transport but is not adequate to capture the features of *Pelosinus* transport. Breakthroughs resulting from the coupled CTRW model perform well at early time and for short control plane distances, especially for *Pelosinus*. However, it fails to match long distance travel and late time concentration. The coupled and the uncoupled CTRW model tend to resemble each other for late-times. In summary, for *Geobacter* which has a pili (twitching) guided motility, none of the three models perform well in matching the real path breakthrough data; and for *Pelosinus* which has a flagella (swimming) guided motility, the coupled CTRW model match real path breakthrough plots well for short control plane distances (i.e., when L is 10 or 20 microns in our study). The fact that none of the models used in this study was able to predict the real breakthrough plots well points to the need of developing more sophisticated modeling tools for studying ensemble transport of bacteria, possibly by constructing random walk processes that also takes correlation structures of jump length and waiting time into consideration.

**Figure 8:**
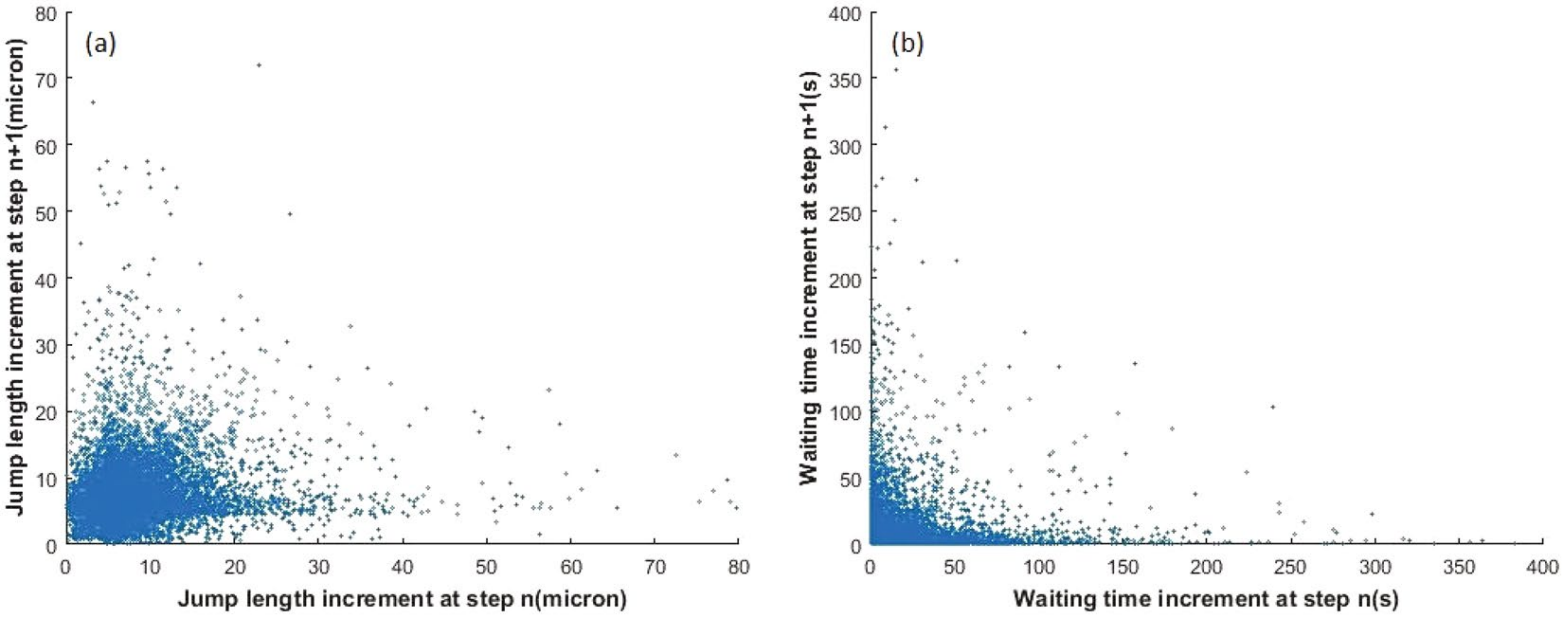
Correlations at step n and step n + 1 for *Pelosinus* for (**a**) jump length and (**b**) waiting time.

Only about 10% of *Geobacter* and 30% of *Pelosinus* passed the first control plane (located at 10 µm from the origin) within 250 seconds. A longer imaging time is required to record more trajectories. The transport features of bacterial transport may not be constant over a time period (as evidenced by Fig. 5 showing varying rates of growth in spread at different times). CTRW and ADE models assume that every bacterium has a finite probability to carry long and short jumps and that each jump is independent from each other. This may, however, not be true. Figure 8 shows correlations of jump length and waiting time increments at step n and step n + 1 for *Pelosinus*. The correlation data was very sparse for *Geobacter* and hence is not presented here. For *Pelosinus*, there are signs of positive correlation in jump lengths and negative correlation in waiting times (a longer waiting time leads to a shorter waiting time in the next step, and vice versa). These correlation structures in jump length and waiting time were not considered in the models presented in this paper, but including them in a more sophisticated future model will likely yield better match with the real breakthrough plots.

## Supplementary information

Supplementary Video

Supplementary Figures

## Data Availability

The datasets generated and/or analyzed during the current study are available from the corresponding author on reasonable request.
