# Supplementary material for: On Modeling Ensemble Transport of Metal Reducing Motile Bacteria
Source: Sci Rep. 2019 Oct 10;9:14638. doi: 10.1038/s41598-019-51271-0 (PMC6787022; doi:10.1038/s41598-019-51271-0)
Supplement: Supplementary file 2 — Supplementary Figures [file 41598_2019_51271_MOESM2_ESM.docx]

**SUPPLEMENTARY FIGURES**

**On Modeling Ensemble Transport of Metal Reducing Motile Bacteria**

Xueke Yang^1^, Rishi Parashar^1*^, Nicole L. Sund^1^, Andrew E. Plymale^2^, Timothy D. Scheibe^3^, Dehong Hu^3^, and Ryan T. Kelly^4^

^1^Division of Hydrologic Sciences, Desert Research Institute, Reno, NV, USA 89512

^2^Energy and Environment Directorate, Pacific Northwest National Laboratory, Richland, WA, USA 99354

^3^Environmental Molecular Sciences Laboratory, Pacific Northwest National Laboratory, Richland, WA, USA 99354

^4^Department of Chemistry and Biochemistry, Brigham Young University, Provo, UT, USA 84602

**
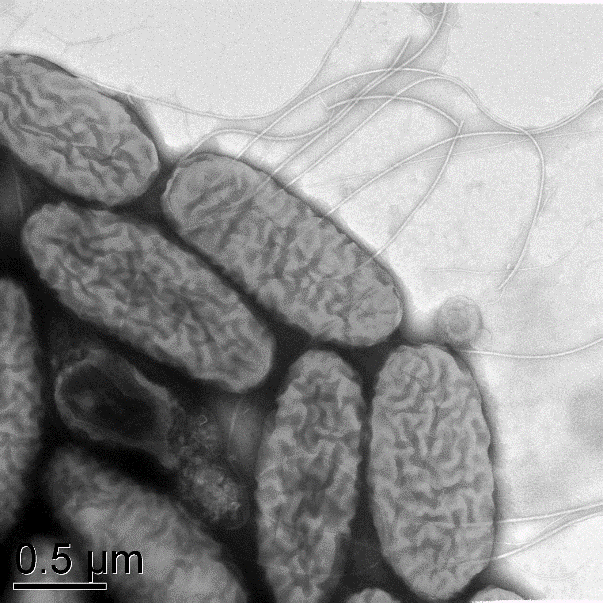
**

**Figure 1: Whole-mount transmission-electron microscopy (TEM) image of *Pelosinus* strain JHL-11 (courtesy Ji-Hoon Lee and Alice Dohnalkova) showing peritrichous flagella.**


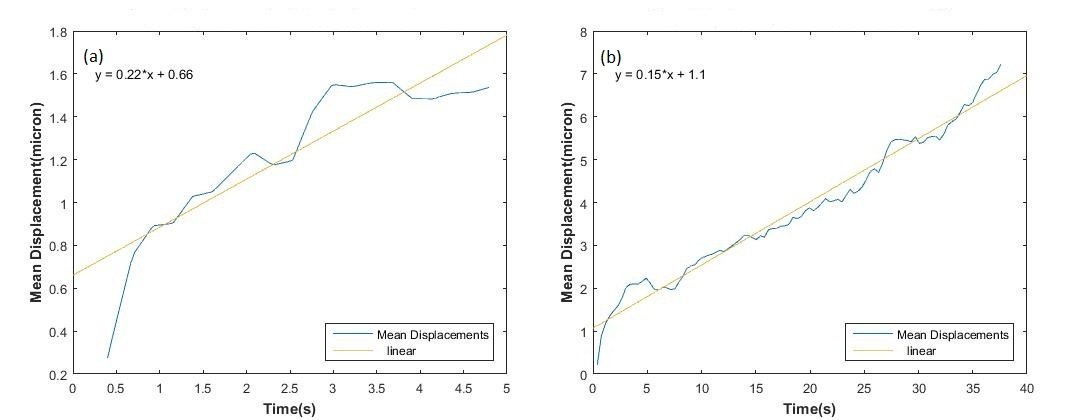


**Figure 2: Mean Displacements over time for a) *Geobacter* and b) *Pelosinus***
